# Relationship between chronotypes and aggression in adolescents: a cross-sectional study

**DOI:** 10.1186/s12888-023-04615-0

**Published:** 2023-03-29

**Authors:** Yuan Wang, Hao Liu, Yan-rong Wang, Jia Wei, Ran-ran Zhao, Jian-qun Fang

**Affiliations:** 1 Mental Health Center, The General Hospital Ningxia Medical University, 750004 Yinchuan, People’s Republic of China; 2 Graduate School, Xi’an International Studies University, 710119 Xi’an, People’s Republic of China; 3 School of Nursing, Ningxia Medical University, 750004 Yinchuan, People’s Republic of China; 4 The Fourth Department of Geriatrics, Hangzhou Seventh People’s Hospital, 310012 Hangzhou, People’s Republic of China; 5 Mental Health Center, the First Hospital of Hebei Medical University, Hebei Technical Innovation Center for Mental Health Assessment and Intervention, 050031 Shijiazhuang, Hebei Province China

**Keywords:** Circadian rhythm, Adolescents, Aggression, Personality traits, Cross-sectional study

## Abstract

**Background:**

This study aimed to investigate the relationship between chronotypes and aggression in adolescents.

**Methods:**

A cross-sectional study was conducted on 755 primary and secondary school students aged 11–16 years in rural areas of Ningxia Province, China. The Chinese version of the Buss-Perry Aggression Questionnaire (AQ-CV) and the Chinese version Morningness-Eveningness Questionnaire (MEQ-CV) were used to assess the aggressive behavior and chronotypes of the study subjects. The Kruskal-Wallis test was then used to compare the differences in aggression among adolescents with different chronotypes, and Spearman correlation analysis to determine the relationship between chronotypes and aggression. Further linear regression analysis was used to investigate the effects of chronotype, personality traits, family environment, and class environment on adolescent aggression.

**Results:**

There were significant differences in chronotypes between different age groups and different sexes. Spearman correlation analysis showed that the MEQ-CV total score was negatively correlated with the AQ-CV total score (*r* = -0.263) and score of each AQ-CV subscale. In Model 1, chronotypes were negatively associated with aggression when controlling for age and sex, and evening-type adolescents might be more likely to exhibit aggressive behavior (b = -0.513, 95% CI: [-0.712, -0.315], *P* < 0.001); in Model 2, the negative association remained after controlling for family and class environment on the basis of Model 1 (b = -0.404, 95% CI: [-0.601, -0.208], *P* < 0.001); and in Model 3, the negative association still existed after controlling for personality traits on the basis of Model 2 (b = -0.383, 95% CI: [-0.577, -0.190], *P* < 0.001).

**Conclusion:**

Compared to morning-type adolescents, evening-type adolescents were more likely to exhibit aggressive behavior. Given social expectations for MT adolescents, adolescents should be actively guided to develop a good circadian rhythm that may be more conducive to their physical and mental development.

## Background

Circadian rhythms (CRs) are 24 h oscillations in physiology, biochemistry, behavior, and other life activities of the body, and this phenomenon is continuously driven by biological clock genes and clock-controlled genes [1]. CRs play a key role in sleep patterns, eating behavior, hormone release, blood pressure, and body temperature regulation [2]. Different circadian phenotypes are generated in different individuals. Influenced by the circadian clock, individuals have a significant subjective preference for their own sleep-wake time [3]. Circadian typology mainly consists of morning type (MT), evening type (ET), and intermediate type (IT). MT subjects prefer to go to bed early and wake up early, and are more energetic during the day; ET subjects prefer to sleep late and wake up late, and generally perform better performance at work in the afternoon or night; IT is in between, and about 60% of adults fall into the IN [4]. CRs have a wide impact on individual psychological activities. Previous studies have shown that ET individuals are more prone to negative emotions such as tension, anxiety, and depression compared to MT and IT individuals [5, 6]. Additionally, ET subjects have a more obvious violent tendency, which is mainly manifested in more frequent verbal aggression, antisocial behavior, retaliation, malice, and irritability and lack of patience in the process of communication with people [7–9].

The definition and classification of aggressive behavior vary in different disciplines and research directions. Buss et al. emphasized target-directed characteristics and behavioral consequences of aggression and believed that aggressive behavior was the result of a combination of impulsivity, irritability, hostility, and anger, and was the individual’s response to transmit harmful stimuli to another organism [10]. There is a significant association between aggressive behavior of different chronotypes and individual personality traits. MT group is more active, easygoing, cooperative, and conscientious, while ET group is more neurotic and extraverted [11]. The occurrence and development of aggression are influenced by many factors, with personality, family environment, and social environment serving as the main contributors and most widely studied [12, 13]. However, no studies have focused on the relationship between chronotype and aggression in Chinese adolescents. Therefore, we aimed to investigate this relationship and thus provide data support for the relationship between chronotype and aggression.

## Methods

### Participants

Primary and secondary schools in rural areas of Ningxia Province in China were selected by simple random sampling (random number table method), and then students who met the inclusion criteria and exclusion criteria were selected from the selected schools by cluster sampling. This study included participants from 18 schools, including 8 junior high schools and 10 primary schools. Students in the same class were defined as a group (typically comprised of 35–45 students). A total of 800 students were selected for a questionnaire survey, and 755 of 800 students completed the questionnaire, with a questionnaire recovery rate of 94.4%. Therefore, the sample size for this study was 755 primary and middle students, with 365 boys and 390 girls. The participants were aged between 11 and 16 years with a mean age of 13.61 ± 1.39 years.

Inclusion criteria were as follows: (1) attending the current school for at least 6 months; (2) aged between 11 and 16 years. The exclusion criteria were as follows: (1) students with vision or hearing impairment; (2) students with learning difficulties; (3) students with diseases that seriously hinder communication; and (4) students with non-standard family backgrounds (e.g., single-parent families).

## Measures

### Chronotypes

Chronotypes were assessed using the Chinese version of Morningness-Eveningness Questionnaire (MEQ-CV) translated and introduced by Zhang Bin et al. in 2006 [14]. The questionnaire consisted of 19 items that measured the subject’s activity in the morning or evening. Its total scores ranged from 16 to 86, with lower total score being considered more ET and higher total scores being considered more MT. Subjects were divided into three groups according to the measured total score: MT 63 to 86 points, IT 50 to 62 points, and ET 16 to 49 points.

### Aggression

In this study, the Chinese version of the Buss-Perry Aggression Questionnaire (AQ-CV) revised by Li et al. was used for the assessment of aggression [15]. The questionnaire included 30 items and 5 subscales (physical aggression, verbal aggression, anger, hostility, and self-aggression). Each item was scored 1–5, representing “extremely uncharacteristic”, “somewhat uncharacteristic”, “neither uncharacteristic nor characteristic”, “somewhat characteristic”, and “extremely characteristic”, respectively. The total score of the questionnaire was the sum of the scores of each subscale, and the score of each subscale was the sum of the scores of the items contained in it. A higher total score on the scale was considered more aggressive.

### Personality

The Eysenck Personality Questionnaire (EPQ) for children was used to assess personality traits [16], with a total of 88 questions. The scale included four subscales: Psychoticism (P), Extraversion (E), Neuroticism (N), and Lying (L). A yes (1 point) or no (0 point) answer to each question was given by the subjects. The standard scores were calculated based on the scores of each subscale and used to analyze the personality traits of the subjects. High P scores indicated psychoticism, while low P scores indicated socialization; high E scores represented extroversion, while low E scores represented introversion; higher N scores suggested neuroticism, while low N scores suggested stability. A high L score indicated dissimulation or fraud, and although L subscale could not reflect an independent personality structure, it was functionally linked to other subscales and represented a stable personality function [17].

### Family environment and class environment

The Family Environment Scale (FES) and a questionnaire named My Class were used to measure the environmental characteristics of the family and society, respectively. The FES checklist consisted of 90 items and contained 10 subscales (cohesion, expressiveness, conflict, independence, achievement orientation, intellectual-cultural orientation, active-recreational orientation, moral-religious emphasis, organization, and control) that measured family relationship, personal growth, and system maintenance [18]. The class environment measurement was performed using Jiang Guanghong’s revised version of the My Class questionnaire, which contained 38 items. The class environment was measured by this questionnaire in terms of five mutually independent dimensions: teacher-student relationship, student-student relationship, order and discipline, competition, and learning burden [19].

### Questionnaire survey process

After communicating with the 18 selected schools, we invited students and their parents or guardians to a classroom where researchers explained the purpose of the current study and the content of the questionnaires. After students and their parents or guardians agreed to participate in the study, a questionnaire survey was administered by trained graduate students majoring in psychology at a medical university, and approximately 40 min were allowed to answer the questionnaires. The questionnaire was checked for completeness by graduate students majoring in psychology, and those with incomplete answers were considered invalid and excluded.

### Statistical analysis

Statistical analysis was performed using SPSS version 24.0. Enumeration data were expressed as N/percentage (%), and the chi-square test or Fisher test was used for comparison between groups. Measurement data conforming to normal distribution were expressed as mean ± standard deviation (SD), and t-test was employed for comparison between groups; measurement data with skewed distribution were expressed as median and quartile [M (P25, P75)], and Mann-Whitney U test was adopted for comparison between groups. The Kruskal-Wallis test was utilized to compare differences in aggression among adolescents with different chronotypes, and the relationship between chronotypes and aggression was analyzed via Spearman correlation analysis. Additionally, the effects of CRs, personality traits, family environment, and class environment on adolescent aggression were investigated by linear regression analysis.

## Results

### Aggression questionnaire scores and chronotypes in adolescents with different demographic characteristics

The Mann-Whitney U test was performed on the total score of the AQ-CV aggression questionnaire and the scores of each subscale for different sexes (boys/girls) and different age groups. As shown in Table 1, the total score of AQ-CV scale and the scores of each subscale in boys (*n* = 365) were higher than those in girls (*n* = 390) (*P* < 0.05). The included adolescents were divided into three age groups: 202 aged 11–12 years, 290 aged 13–14 years and 263 aged 15–16 years. As shown in Table 2, significant differences among three age groups were identified in the scores of self-aggression, hostility, anger, verbal aggression subscales and total score of AQ-CV aggression questionnaire (*P* < 0.05), but not in the scores of physical aggression subscale (*P* > 0.05).

**Table 1:** Comparison of aggression questionnaire scores and chronotypes among adolescents of different sexes

Variables	Boy (*n* = 365)	Girl (*n* = 390)	Statistics	*p*
AQ-CV total score	68.0[46.0,84.0]	57.0[42.0,75.0]	3.8	< 0.001
Self-aggression score	12.0[6.0,14.0]	9.0[6.0,13.0]	2.8	0.005
Hostility score	16.0[10.0,20.0]	14.0[10.0,18.0]	3.4	< 0.001
Anger score	14.0[9.0,18.0]	12.0[8.0,17.0]	2.7	0.007
Verbal aggression score	10.0[7.0,12.0]	9.0[7.0,11.0]	3.4	< 0.001
Physical aggression score	16.0[10.0,21.0]	13.0[8.0,17.0]	5.2	< 0.001
Chronotypes			13.0	0.002
Evening-type(%)	32(8.8)	24(6.2)		
Intermediate-type(%)	194(53.2)	167(42.8)		
Morning-type (%)	139(38.1)	199(51.0)		

Note: Measurement data were presented using median and quartile [M (P25, P75)], and enumeration data were presented using N/percentage (%). AQ-CV, Chinese version of the Buss-Perry Aggression Questionnaire

**Table 2:** Comparison of aggression questionnaire scores and chronotypes among adolescents of different age groups

Variables	Aged 11–12 (*n* = 202)	Aged 13–14 (*n* = 290)	Aged 15–16 (*n* = 263)	Statistics	*p*
AQ-CV total score	67.0[49.0,85.0]	60.0[41.0,79.0]	60.0[44.0,79.0]	10.3	0.006
Self-aggression score	12.0[7.0,15.0]	9.0[6.0,14.0]	10.0[7.0,14.0]	12.2	0.002
Hostility score	16.0[12.0,20.0]	14.0[9.0,19.0]	14.0[10.0,19.0]	10.2	0.006
Anger score	14.0[9.0,18.0]	12.0[8.0,17.0]	13.0[8.0,17.0]	8.5	0.014
Verbal aggression score	10.0[7.0,12.0]	9.0[7.0,11.0]	9.0[7.0,11.0]	8.4	0.015
Physical aggression score	15.0[10.0,20.0]	14.0[8.0,19.0]	14.0[9.0,19.0]	3.9	0.139
Chronotypes				19.0	< 0.001
Evening-type(%)	12(5.9)	15(5.2)	29(11.0)		
Intermediate-type (%)	95(47.0)	125(43.1)	141(53.6)		
Morning-type (%)	95(47.0)	150(51.7)	93(35.4)		

Note: Measurement data were presented using median and quartile [M (P25, P75)], and enumeration data were presented using N/percentage (%). AQ-CV, Chinese version of the Buss-Perry Aggression Questionnaire

The Chi-square test was used to compare differences in the distribution of chronotypes among adolescents of different ages and sexes. The results showed that there were significant differences in chronotypes between adolescents of different age groups (*P* = 0.002, Table 1) and adolescents of different sexes (*P* < 0.001, Table 2).

### Spearman correlation analysis of chronotypes and aggression in adolescents

**Fig. 1:**
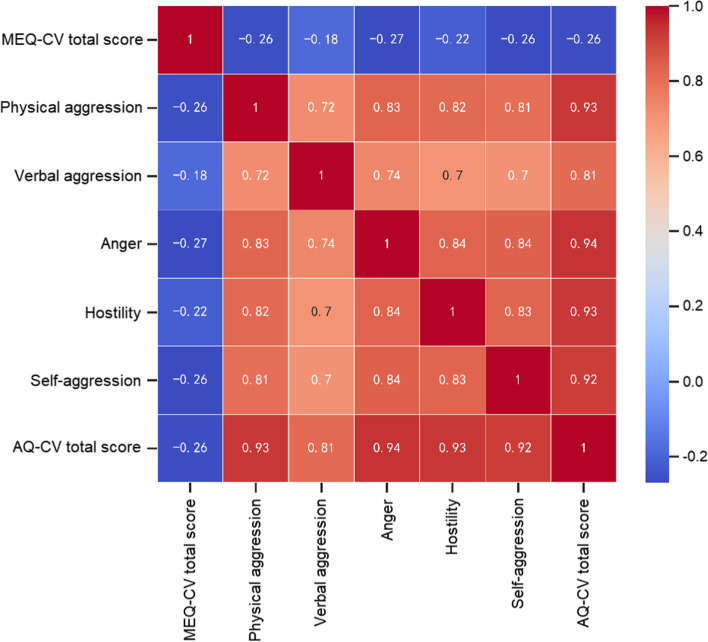
Spearman correlation analysis of chronotypes and aggression in adolescents. Values were correlation coefficient (r); red indicated positive correlation and blue indicated negative correlation. AQ-CV, Chinese version of the Buss-Perry Aggression Questionnaire; MEQ-CV, Chinese version of Morningness-Eveningness Questionnaire

Spearman correlation analysis was used to analyze the correlation of MEQ-CV total score with AQ-CV total score and the scores of each subscale. The results showed (Fig. 1) that the MEQ-CV total score was negatively correlated with the AQ-CV total score (*r* = -0.263) and scores of physical aggression (*r* = -0.258), verbal aggression (*r* = -0.179), anger (*r* = -0.268), hostility (*r* = -0.215), and self-aggression (*r* = -0.265) subscales.

### Comparison of aggression in adolescents with different chronotypes

The Kruskal-Wallis test was used to compare aggressive behavior among adolescents with different chronotypes. As shown in Table 3, ET adolescents had significantly higher physical aggression score (F = 43.1, *P* < 0.001), verbal aggression score (F = 23.4, *P* < 0.001), anger score (F = 44.9, *P* < 0.001), hostility score (F = 31.4, *P* < 0.001), self-aggression score (F = 44.3, *P* < 0.001) and AQ-CV total score (F = 44.3, *P* < 0.001) compared with the IT and MT adolescents (Table 3).

**Table 3:** Comparison of aggression in adolescents with different chronotypes

Variables	Evening-type (*n* = 56)	Intermediate-type (*n* = 361)	Morning-type (*n* = 338)	F	*p*
Physical aggression score	16.0[10.0,19.0]	16.0[11.0,21.0]	12.0[8.0,17.0]	43.1	< 0.001
Verbal aggression score	10.0[7.0,11.0]	10.0[8.0,12.0]	9.0[7.0,11.0]	23.4	< 0.001
Anger score	15.0[10.0,18.0]	15.0[9.0,18.0]	11.0[7.0,15.0]	44.9	< 0.001
Hostility score	16.0[12.0,21.0]	16.0[10.0,21.0]	13.0[9.0,17.0]	31.4	< 0.001
Self-aggression score	12.0[9.0,15.0]	12.0[7.0,15.0]	9.0[5.0,12.0]	44.3	< 0.001
AQ-CV total score	69.0[51.0,83.0]	70.0[48.0,86.0]	54.0[38.0,72.0]	44.3	< 0.001

Note: Measurement data were presented using median and quartile [M (P25, P75)]. AQ-CV, Chinese version of the Buss-Perry Aggression Questionnaire

### Linear regression analysis between chronotypes and aggression in adolescents

In Model 1 (chronotypes + age, sex), a negative correlation was found between chronotypes and aggressive behavior, and evening-type adolescents might be more likely to exhibit aggressive behavior (b = − 0.513, 95% CI: [− 0.712, − 0.315], *P* < 0.001). In Model 2 (Model 1 + family environment, class environment), chronotype remained negatively associated with aggression (b = -0.404, 95% CI: [-0.601, -0.208], *P* < 0.001). Conflict in the family environment was positively associated with aggression (b = 1.228, 95% CI: [0.189, 2.268], *P* = 0.021), while cohesion (b = − 1.340, 95% CI: [− 2.490, − 0.190], *P* = 0.022) and organization (b = − 1.263, 95% CI: [− 2.405, − 0.120], *P* = 0.030) were negatively associated with aggression. Aggression was positively associated with competition in the class environment (b = 1.294, 95% CI: [0.753, 1.835], *P* < 0.001), and was negatively associated with teacher-student relationship (b = − 0.642, 95% CI: [− 1.071, − 0.213], *P* = 0.003). In Model 3 (Model 2 + personality traits), there was still a negative association between chronotypes (b = -0.383, 95% CI: [-0.577, -0.190], *P* < 0.001). However, N in personality characteristics was positively associated with aggression (b = 0.575, 95% CI: [0.078, 1.072], *P* = 0.023) (Table 4).

**Table 4:** Linear regression analysis between chronotypes and aggression in adolescents

Variables	Model 1		Model 2		Model 3	
	*P* value	b (95%CI)	*P* value	b (95%CI)	*P* value	b (95%CI)
Sex	0.001	-5.681(-9.024,-2.339)	0.007	-4.448(-7.692,-1.204)	0.024	-3.691(-6.884,-0.498)
Age	0.050	-1.159(-2.319,0.000)	0.013	-1.431(-2.555,-0.308)	0.008	-1.505(-2.612,-0.399)
chronotypes	**<0.001**	-0.513(-0.712,-0.315)	**＜0.00**	-0.404(-0.601,-0.208)	**＜0.001**	-0.383(-0.577,-0.190)
Cohesion	NA	NA	**0.022**	-1.340(-2.490,-0.190)	0.021	-1.334(-2.465,-0.202)
Expressiveness	NA	NA	0.429	0.516(-0.765,1.798)	0.554	0.380(-0.878,1.637)
Conflict	NA	NA	**0.021**	1.228(0.189,2.268)	0.210	0.666(-0.375,1.706)
Independence	NA	NA	0.749	-0.179(-1.274,0.917)	0.204	-0.706(-1.796,0.384)
Achievement orientation	NA	NA	0.821	0.123(-0.942,1.188)	0.616	-0.269(-1.323,0.784)
Intellectual-cultural orientation	NA	NA	0.979	0.017(-1.215,1.248)	0.950	-0.039(-1.248,1.170)
Active-recreational orientation	NA	NA	0.872	-0.093(-1.230,1.043)	0.825	-0.126(-1.243,0.991)
Moral-religious emphasis	NA	NA	0.989	-0.008(-1.151,1.134)	0.807	-0.140(-1.264,0.984)
Organization	NA	NA	**0.030**	-1.263(-2.405,-0.120)	0.009	-1.513(-2.643,-0.383)
Control	NA	NA	0.353	0.561(-0.623,1.744)	0.791	0.158(-1.016,1.333)
Teacher-student relationship	NA	NA	**0.003**	-0.642(-1.071,-0.213)	0.006	-0.603(-1.030,-0.176)
Student-student relationship	NA	NA	0.862	0.050(-0.516,0.616)	0.769	0.083(-0.475,0.642)
Order and discipline	NA	NA	0.181	-0.312(-0.769,0.146)	0.418	-0.186(-0.637,0.265)
Competition	NA	NA	**＜0.001**	1.294(0.753,1.835)	**＜0.001**	1.177(0.643,1.710)
Learning burden	NA	NA	0.157	0.389(-0.150,0.929)	0.190	0.356(-0.176,0.887)
E personality	NA	NA	NA	NA	0.146	0.531(-0.185,1.247)
N personality	NA	NA	NA	NA	**0.023**	0.575(0.078,1.072)
P personality	NA	NA	NA	NA	0.886	-0.052(-0.774,0.669)
R^2^	0.055		0.161		0.196	

Model l = chronotypes + age, sex; Model 2 = Model l + family environment (cohesion, expressiveness, conflict, independence, achievement orientation, intellectual-cultural orientation, active-recreational orientation, moral-religious emphasis, organization, and control), class environment (teacher-student relationship, student-student relationship, order and discipline, competition, and learning burden); Model 3 = Model 2 + personality traits (E, N, P); *OR *Odds confidence ratio, *95% CI *95% confidence interval, *NA *Apply not, *E *Extraversion, *N *Neuroticism, *P *Psychoticism

## Discussion

Individuals of different chronotypes tend to have different psychological activities. Compared with MT subjects, ET subjects are more likely to experience negative emotions such as depression and aggressive behavior [9]. However, there is a lack of data on the relationship between chronotypes and aggression in adolescents. In this study, we investigated the relationship between chronotypes and adolescent aggression through a questionnaire survey among 755 students from primary and secondary schools in rural areas of Ningxia Province, China.

There were significant differences in chronotypes as well as aggressive behaviors among adolescents of different ages and adolescents of different sexes in this study. Consistent with a previous study [20], we found boys were more likely to be ET and girls were more likely to be MT. Chronotypes change correspondingly with age. For example, MT is more frequent during childhood, while adolescents, young adults, and middle age are more inclined to ET [21, 22]. In the results of the study, boys showed greater aggression compared to girls. This difference may be related to the different emotional regulation of adolescents of different sexes; girls generally have more prosocial emotion expressions, while boys are more likely to externalize their emotions [23, 24].

There was a significant association between chronotypes and aggression in this study. Roughly consistent with previous findings [25, 26], we found ET adolescents were more aggressive, mainly manifested in physical aggression, verbal aggression, anger, and hostility. Some researchers have shown that aggressive behavior is associated with CRs and clock genes, which possibly affect the levels of neurotransmitters including dopamine, serotonin, and norepinephrine [27–29]. This study showed that chronotypes were negatively associated with aggression after controlling for demographic characteristics and psychosocial variables. Previous studies have shown that ET type has a stronger correlation with psychological factors and mental disorders than other types, and the effects of sleep deprivation on human behavior and emotions may explain such a difference [30]. Sleep deprivation is a stressor that may increase the risk of suicide by impairing cognitive judgment or impulse control, increasing irritability and physical problems, or lowering negative motor response thresholds [31]. It has been shown that sleep-deprived individuals are more likely to have aggressive behavior [32], and ET individuals are prone to being deprived of sleep and thus their emotions and personality are affected [33]. The above findings may explain why ET individuals are more impulsive and aggressive [34]. However, in Figueiredo’s study, inconsistent with our findings, mainly in that it investigated the relationship between time type, classroom behavior and school performance in 140 Portuguese children and found that early morning type children showed greater oppositional behavior, motor restlessness and impulsivity than night type children [35].

Aggression was positively correlated with competition and was negatively correlated with teacher-student relationship. The reason may be that intense competition and negative interpersonal interaction increase students’ stress, so they tend to display risky behaviors such as rebellion. In other words, a good teacher-student relationship and peer relationships can have a positive guiding effect on the behavioral and psychological development of adolescents and reduce the occurrence of aggressive behavior [36, 37]. After controlling for family environment, class environment and personality traits, the negative association between chronotypes and aggression still exists. Consistently, a study found that aggression remained associated with chronotypes after controlling for psychosocial factors, and thus neurobiological factors are thought to contribute to the effect of chronotypes on aggression [38]. Murray-Close D et al. demonstrated that there was a correlation between CRs of cortisol and physical aggression in abused children and age-matched controls (6–12 years) [39], and other related studies [40–42] all suggested that some specific forms of aggression were associated with different CRs of cortisol secretion. Both the CRs and autonomic nervous system regulate many hormones, and there is substantial evidence supporting the effects of various hormones on aggression in animals and humans [43, 44]. CRs and autonomic nerves clearly influence neuromodulatory processes, and neuromodulation of aggressive behavior interacts with neuromodulation of CRs; current aggressive episodes have been shown to entrain CRs [45]. To conclude, CRs, autonomic and neuroendocrine systems are highly connected, and each system influences emotional states and stressful behaviors [46].

There was an inverse association between chronotypes and aggression after controlling for personality traits on the basis of Model 2. MT individuals show behavioral self-control, and tend to reach out to the outside world in a respectful and cooperative manner and manifest themselves in a formal and appropriate way in social situations [47]. The social expectation of students is MT chronotype. MT individuals give others a more positive impression, and they are more responsible for self-esteem and self-control [11]. By contrast, ET subjects tend to be more creative and adventurous, ready to convert and recast whatever they encounter. As for behavioral style, ET individuals are likely to act in an independent, nonstandard manner and resist following traditional standards [48]. It has been shown that ET group has significantly higher scores in aggressive-hostility and impulsive sensation seeking than the MT group and the ET group has a more significant tendency to violent impulses than the MT and IT groups, which are basically consistent with the results of this study [49].

This study still has several limitations. (1) This is a cross-sectional study with the sample size mainly from primary and secondary schools in rural areas of Ningxia Province, China, so whether the findings are applicable to adolescents in other areas still needs further verification. (2) This study did not formulate other screenings were performed to rule out psychiatric diagnosis in the sample. (3) The circadian rhythm type of the participants was only assessed by a questionnaire and no relevant laboratory tests (e.g., actigraphy and cortisol levels) were performed on the participants. In future studies, we will include a larger sample size for more regions.

## Conclusion

This study provides evidence to demonstrate the association between chronotypes and aggression. ET adolescents were more likely to exhibit aggressive behavior relative to MT adolescents. MT adolescents may be more in line with society’s expectations. Adolescents should be actively guided to develop a good CR which may be more conducive to their physical and mental development. It is suggested that their sleep-wake phase be advanced and their chronotype be changed closer to the morning-type.

## Data Availability

The datasets generated and/or analysed during the current study are not publicly available due to privacy but are available from the corresponding author on reasonable request.
